# A Phosphorus(V)‐Centered Porphyrin Having Redox‐ and Air‐Stable Axial P–H Bonds

**DOI:** 10.1002/chem.202502215

**Published:** 2025-09-07

**Authors:** Shintaro Ishida, Yoshiki Ota, Nozomu Kuwabara, Takuroh Hatakeyama, Takeaki Iwamoto

**Affiliations:** ^1^ Department of Chemistry Graduate School of Science Tohoku University Aoba‐ku Sendai 9808578 Japan

**Keywords:** cations, phosphorus heterocycles, porphyrinoids, redox chemistry

## Abstract

Phosphorus(V)‐centered porphyrins (P(V)‐porphyrins) are an important class of functional dyes in many fields of research, and axial ligands on the phosphorus atom affect the electronic properties of P(V)‐porphyrins and add functions. Herein, we report on the synthesis and characterization of a hitherto unknown P(V)‐porphyrin having hydrogen atoms as axial ligands (**1** ^+^ **·**PF_6_^–^, PF_6_^–^ is a counter anion). Synthesis of **1** ^+^ **·**PF_6_^–^ was achieved by treatment of dichloro‐derivative (**2** ^+^ **·**Cl^–^) with LiAlH_4_ followed by AgPF_6_ via hydride reduction accompanied by one‐electron reduction and one‐electron oxidation. The porphyrin core of **1** ^+^ **·**PF_6_^–^ is nonplanar but considerably ruffled. The cyclic voltammogram of **1** ^+^ **·**PF_6_^–^ exhibits two reversible waves, which indicates redox couples among **1** ^+^, neutral radical **1^•^**, and **1** ^–^. Compound **1** ^+^ **·**PF_6_^–^ is tolerant toward air despite its PH_2_ moiety due to the energetically low‐lying HOMO (highest occupied molecular orbital) and σ(P–H) orbitals.

## Graphical abstract

Phosphorus‐centered porphyrins are important functional dyes. Herein, we report the synthesis and characterization of a phosphorus(V) porphyrin complex with hydrogen atoms as axial ligands (**1** ^+^ **·**PF_6_^–^). **1** ^+^ **·**PF_6_^–^ is air‐stable. Cyclic voltammetry of **1** ^+^ **·**PF_6_^–^ exhibits two reversible waves, which indicates sequential redox couples among cationic, neutral open‐shell, and anionic species.

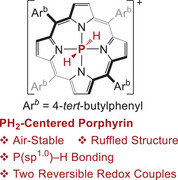

## 1 Introduction

Phosphorus(V)‐centered porphyrins (P(V)‐porphyrins) are one of the representative main group element‐centered porphyrin complexes and occupy an important class of functional dyes in materials sciences.^[^ ^1^, ^2^, ^3^, ^4^, ^5^, ^6^, ^7^, ^8^, ^9^, ^10^, ^11^, ^12^, ^13^, ^14^, ^15^, ^16^, ^17^, ^18^, ^19^ ^]^ For instance, singlet oxygen is generated effectively by irradiating visible light to P(V)‐porphyrins, which are pharmaceutical candidates for photodynamic therapy.^[^ ^20^, ^21^, ^22^, ^23^, ^24^, ^25^, ^26^, ^27^, ^28^, ^29^, ^30^ ^]^ P(V)‐porphyrin derivatives also act as electron acceptors for photovoltaic devices.^[^ ^31^, ^32^ ^]^ In P(V)‐porphyrins, axial ligands are an essential part of their functions. However, those reported so far remain limited to (1) halogens (F, Cl, and Br), (2) oxygen and nitrogen functional groups, and (3) simple alkyl groups (methyl, ethyl, and phenyl).^[^ ^33^, ^34^, ^35^, ^36^, ^37^, ^38^, ^39^, ^40^, ^41^, ^42^, ^43^, ^44^, ^45^, ^46^ ^]^ Hydrogen is the smallest element, and its electronegativity (2.20, based on the Pauling scale) is comparable to that of phosphorus (2.19) and significantly smaller than those of carbon (2.55), nitrogen (3.04), oxygen (3.44), and halogens (2.96–3.98). Therefore, the structure and the electronic properties of hitherto unknown PH_2_‐porphyrin are of particular interest. Moreover, most P–H bonds are readily functionalized by various methods involving hydrophosphination. Thus, axial P–H bonds should expand the chemical space of P(V)‐porphyrins. Herein, we report on the synthesis, isolation, and properties of a cationic PH_2_‐porphyrin.

## 2 Results and Discussion

### 2.1 Synthesis and Molecular Structure

**Scheme 1:**
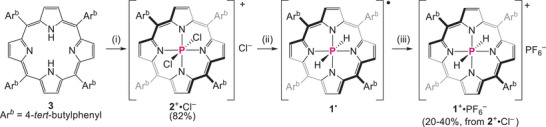
Synthesis of **1** ^+^ **·**PF_6_^–^. Conditions: (i) POCl_3_ (excess), pyridine, reflux, 24 hours. (ii) LiAlH_4_ (4.0 equiv.), THF, rt. (iii) AgPF_6_ (1.2 equiv.), rt, CH_2_Cl_2_.

We synthesized dichlorophosphorus(V) porphyrin **2** ^+^ **·**Cl^–^ bearing four 4‐*tert*‐butylphenyl groups at the meso‐positions as a precursor of the PH_2_‐porphyrin. The reaction of the free‐base porphyrin **3** ^[^ ^47^ ^]^ with phosphorus(V) oxychloride in refluxing pyridine afforded **2** ^+^ **·**Cl^–^ in 82% yield (Scheme 1). When **2** ^+^ **·**Cl^–^ was treated with four equiv of LiAlH_4_ in THF, the color of the solution turned immediately from green to dark yellow. As the resulting solution exhibited an intense EPR signal (Figure S12) and no proton signals due to the porphyrin ring in the ^1^H NMR spectrum, one electron reduction of **2** ^+^ **·**Cl^–^ by LiAlH_4_ would be associated with the desired hydride reduction to generate neutral radical **1^•^**.^[^ ^46^, ^48^ ^]^ After the removal of the residual aluminum hydride species by filtration through a Celite pad, the volatiles of the filtrate were evaporated in vacuo. The solvent was exchanged to dichloromethane, and insoluble materials were filtered off through a celite pad. Then, the dichloromethane solution of the residue was treated with AgPF_6_ for one‐electron oxidation of **1^•^**. Silica gel column chromatography of the crude product and the subsequent recrystallization afforded pure **1** ^+^ **·**PF_6_^–^ as a blue powder. Yields are ranging from 20 to 40% based on **2** ^+^ **·**Cl^–^. The reason for the low reproducibility of yields is unclear despite efforts such as reaction monitoring and using fresh LiAlH_4_. NMR spectra of the reaction mixture suggest that the decomposition of the porphyrin core partly proceeds to form inseparable byproducts during the reactions (ii) and (iii) in Scheme 1, and the amount of byproducts varied in runs with almost identical conditions. The structure of **1** ^+^ **·**PF_6_^–^ was determined by NMR spectroscopy, mass spectrometry, and elemental analysis. **2** ^+^ **·**Cl^–^ is hardly soluble in aromatic solvents, while **1** ^+^ **·**PF_6_^–^ is soluble in benzene‐*d*_6_.

**Figure 1:**
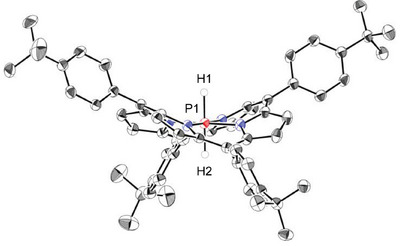
ORTEP of **1** ^+^ **·**PF_6_^–^ with thermal ellipsoids at 50% probability. The solvent molecules, the counterion PF_6_^–^, and hydrogen atoms attached to carbon atoms are omitted for clarity. Crystallographically independent two **1** ^+^ molecules exist in the asymmetric unit, and one of them is shown. For details, see Figures S16 and S17 in the Supporting Information. Selected bond lengths (Å): P1–N1 1.839(3), P1–N2 1.845(3), P1–N3 1.851(3), P1–N4 1.856(3).

The molecular structure of **1** ^+^ **·**PF_6_^–^ in the solid state determined by single‐crystal X‐ray diffraction analysis (sc‐XRD) is shown in Figure 1.^[^ ^49^ ^]^ The hydrogen atoms on the phosphorus atom were found from the difference Fourier map. The averaged P–N distance in **1** ^+^ **·**PF_6_^–^ [1.848(3) Å] is longer than that of **2** ^+^ **·**Cl^–^ [1.812(2) Å] (Figure S15). The observed bond elongation would result from the increased electron repulsion between P and N atoms; the phosphorus atom becomes electron‐rich by replacing axial ligands from chlorine atoms to hydrogen atoms. The six‐coordinate P1 atom adopts an octahedral geometry; the P1 atom was located on the least‐squares plane of the four nitrogen atoms (N_4_‐plane). The porphyrin core of **1** ^+^ is considerably ruffled; the *meso*‐carbon atoms are located 0.90–1.04 Å above and below the N_4_‐plane. The observed large deformation from the planar porphyrin ring is frequently found in the reported P(V)‐porphyrins, which is mainly owing to the relatively short lengths of P–N bonds.^[^ ^50^, ^51^ ^]^

**Figure 2:**
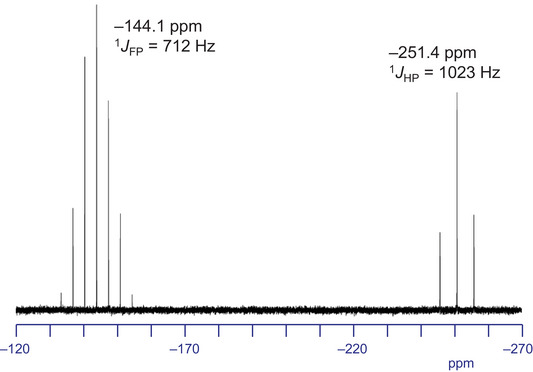
^31^P NMR spectrum of **1** ^+^ **·**PF_6_^–^ in CDCl_3_ at 295 K.

**Figure 3:**
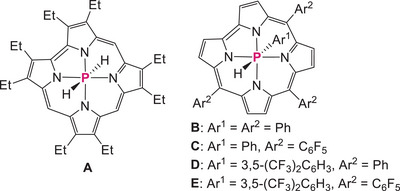
Reported phosphorus(V) corrole complexes having P–H bonds.

The ^31^P NMR spectrum of **1** ^+^ **·**PF_6_^–^ exhibited a triplet signal of the **1** ^+^ at –251.4 ppm (^1^ *J*_HP_ = 1023 Hz) together with a septet signal of the PF_6_^–^ anion (–144.1 ppm, ^1^ *J*_FP_ = 712 Hz) (Figure 2). As the upfield‐shifted signal appeared as a singlet in the ^31^P{^1^H} (proton decoupling) NMR spectrum (Figure S10), this signal is assignable to the PH_2_ moiety of **1** ^+^. The ^1^H NMR spectrum of **1** ^+^ **·**PF_6_^–^ in CDCl_3_ showed a doublet for the PH_2_ unit at –2.19 ppm (^1^ *J*_PH_ = 1023 Hz) which is considerably high‐field shifted from a typical range of those of five‐ and six‐coordinate phosphorus compounds (4‐9 ppm),^[^ ^52^, ^53^, ^54^, ^55^, ^56^, ^57^ ^]^ as the axial hydrogen atoms of **1** ^+^ are located in the shielding area of the porphyrin π‐plane. Similar high‐field shifts have been reported in neutral phosphorus(V) corrole complexes **A‐E** (Figure 3, –2.66 to –3.31 ppm).^[^ ^58^, ^59^ ^]^ The ^1^ *J*_PH_ value of **1** ^+^ is larger than five‐ and six‐coordinate PH compounds (680‐962 Hz),^[^ ^53^, ^54^, ^55^, ^56^, ^57^, ^58^, ^59^ ^]^ which suggests a high s‐character of the phosphorus bonding orbitals of the P–H bonds. The IR spectrum of **1** ^+^ **·**PF_6_^–^ in a KBr pellet exhibited an unsymmetrical stretching of H–P–H moiety [v_as_(PH_2_)] band at 2360 cm^−1^ (Figure S13). The UV‐vis spectrum of **1** ^+^ **·**PF_6_^–^ in THF shows a Soret band (451 nm) and Q bands (587 and 633 nm) (Figure S14), which are slightly bathochromically shifted compared with those of **2** ^+^ **·**Cl^–^ in the same condition [448 nm (Soret band) and 575 and 622 nm (Q‐bands)].

**Figure 4:**
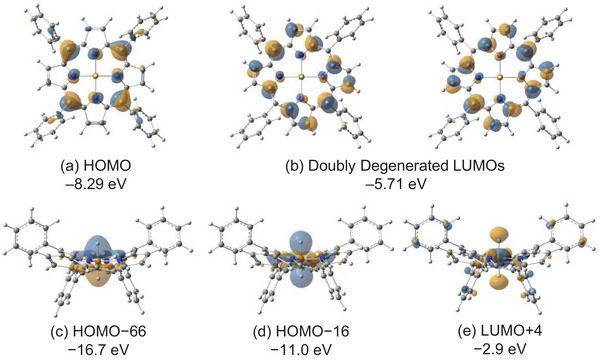
Selected Kohn‐Sham orbitals of **1m** ^+^.

To obtain insight into the bonding character and reactivity of the six‐coordinate PH_2_ unit in **1** ^+^ **·**PF_6_^–^, theoretical studies were carried out using a tetraphenyl derivative (**1m** ^+^) as a model compound.^[^ ^60^, ^61^, ^62^ ^]^ The optimized structure of **1m** ^+^ at the B3PW91‐D3/6–31 + G(d) level of theory reproduced the highly ruffled structure of **1** ^+^ obtained by sc‐XRD. ^1^H and ^31^P NMR chemical shifts of **1m** ^+^ [GIAO‐B3PW91/6–311 + G(2df,p)] are calculated to be –2.60 ppm (^1^ *J*_PH_ = 969 Hz) and –225.0 ppm for the PH_2_ moiety, which agrees with the above‐discussed experimental values. Natural bond orbital (NBO) analysis of **1m** ^+^ indicates that the P–H bonds consist of bonding orbitals with a high s‐character on the phosphorus atom (sp^1.0^).^[^ ^63^ ^]^ The P–H bonds are nonpolar based on the contributions of the phosphorus and hydrogen orbitals (P: 54%, H: 46%). The Mayer bond order (MBO) analysis indicates that the P–H bonds are single bonds (MBO values: 0.94),^[^ ^64^ ^]^ while the small MBO values of the P–N bonds (0.20 each) are suggestive of two sets of three‐center‐four‐electron (3c‐4e) bonds. As shown in Figure 4, the frontier Kohn‐Sham (KS) HOMO (–8.29 eV) and doubly degenerated LUMOs (lowest unoccupied molecular orbitals) (–5.71 eV) of **1m** ^+^ (Figure 4) are π‐ and π*‐orbitals of the porphyrin ring. The PH_2_ σ‐orbitals (HOMO‐16 and HOMO‐66) lie energetically very low (–11.0 and –16.7 eV).

### 2.2 Redox and Reactivity

**Figure 5:**
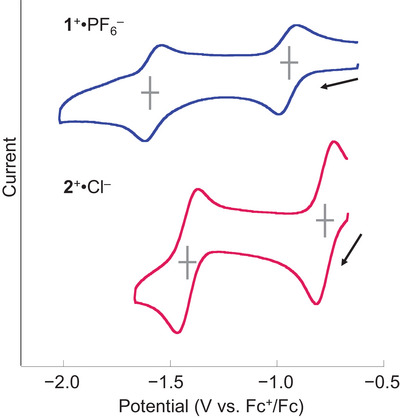
Cyclic voltammograms recorded for **1** ^+^·PF_6_^–^ and **2^+^** ·**Cl^–^** in THF at room temperature. The redox potentials are referenced to the ferrocene/ferrocenium ion couple. Conditions: scan rate 100 mV s^−1^, concentration of P(V)‐porphyrins ca. 0.5 mM, supporting electrolyte Bu_4_N^+^PF_6_^–^(0.1 M).

**1** ^+^ **·**PF_6_^–^ exhibited two reversible reductive waves in THF, versus the ferrocene/ferrocenium (Fc/Fc^+^) couple as shown in Figure 5, and the first and second half‐wave potentials (*E*_1/2_^ox^) are –0.94 V and –1.58 V, respectively. These reductive waves were shifted negatively by 0.16 V from those of **2** ^+^ **·**Cl^–^ (*E*_1/2_^ox^: –0.78 V and –1.42 V). This shift could arise from the less electronegative character of hydrogen atoms compared to chlorine atoms. The electrochemical analysis indicates a two‐step redox system among cationic **1** ^+^, neutral radical **1^•^**, and anionic **1** ^–^. According to the redox properties of **1** ^+^ **·**PF_6_^–^, we carried out the chemical reduction of **1** ^+^ **·**PF_6_^–^. When **1** ^+^ **·**PF_6_^–^ was reduced by KC_8_ (1 equiv) in THF at room temperature, the color of the solution turned brown. The resulting solution showed an intense EPR signal (Figure S12). The black solid was obtained after the removal of volatiles and the subsequent extraction with hexane. The C_6_D_6_ solution of the thus obtained black solid exhibited no ^1^H NMR signals of **1** ^+^ and a small amount of free‐base porphyrin. The treatment of the black solid with AgPF_6_ (1 equiv) in dichloromethane at room temperature regenerated **1** ^+^ **·**PF_6_^–^ in 72% yield from the initial amount of **1** ^+^ **·**PF_6_^–^ based on the ^1^H NMR integrals. These results demonstrated the chemical interchange between **1** ^+^ **·**and **1^•^**. Isolation of **1^•^** from the reaction mixture has been unsuccessful despite efforts. Radical **1^•^** is not tolerant toward silica gel and alumina column chromatography. Recrystallization of **1^•^** did not improve its purity.

Treatments of **1** ^+^ **·**PF_6_^–^ with *n*‐butyllithium or triethylamine underwent one‐electron reduction to generate a mixture containing **1^•^**. The desired deprotonation and the subsequent transformation of the PH_2_ moiety have been unsuccessful. We examined substitution reactions of **1** ^+^ **·**PF_6_^–^ using carbon electrophiles. However, **1** ^+^ **·**PF_6_^–^ remained unreacted in benzyl bromide and allyl bromide after heating at 60 °C for two days. Hydrophosphinations of **1** ^+^ **·**PF_6_^–^ with styrene and benzaldehyde did not proceed owing to the nonpolar P–H bonds. In contrast to common primary phosphines, **1** ^+^ **·**PF_6_^–^ in the solid state is tolerant toward oxygen despite its PH_2_ unit. The salt of **1** ^+^ **·**PF_6_^–^ is bench‐stable for at least two months. The remarkable stability of **1** ^+^ **·**PF_6_^–^ toward oxygen can be explained by the low energy levels of HOMO and P–H bonding orbitals, which suppresses an electron transfer from **1** ^+^ to oxygen to generate **1** ^2+^.^[^ ^65^ ^]^ The generation of the phosphadioxirane ring via ^1^O_2_ is also diminished by the six‐coordination of the phosphorus atom.^[^ ^66^ ^]^

## 3 Conclusion

In conclusion, a P(V)‐porphyrin complex bearing two hydrogen atoms as axial ligands was synthesized and isolated as **1** ^+^ **·**PF_6_^–^. In the solid state, the six‐coordinate phosphorus atom in **1** ^+^ adopts an octahedral geometry, and the porphyrin core has a remarkably ruffled structure as well as reported P(V)‐porphyrins. The computational studies of model compound **1m** ^+^ suggested that the six‐coordination of the phosphorus atom in **1^+^** consists of two P(sp^1.0^)–H bonds and two 3c‐4e N–P–N bonds. Cyclic voltammetry of **1** ^+^ **·**PF_6_^–^ exhibits two reversible reduction waves, indicating redox couples among **1** ^+^, **1^•^**, and **1** ^–^. Compound **1** ^+^ **·**PF_6_^–^ is air‐stable despite its PH_2_ moiety owing to the energetically low‐lying HOMO and σ(P–H) orbitals.

## Supporting Information

The authors have cited additional references in the Supporting Information.^[^ ^47^, ^67^, ^68^, ^69^, ^70^, ^71^, ^72^, ^73^, ^74^ ^]^ Experimental details, synthetic procedures of **1** ^+^ **·**PF_6_^–^ and **2** ^+^ **·**Cl^–^, NMR, IR, EPR, and UV‐vis spectra of the products, and supplemental data of the theoretical studies are described in the Supporting Information.

## Conflict of Interest

The authors declare no conflict of interest.

## Supporting information



Supporting Information

Supporting Information

Supporting Information

## Data Availability

The data of this article are available in the Supporting Information.
