# Supplementary material for: A Phosphorus(V)‐Centered Porphyrin Having Redox‐ and Air‐Stable Axial P–H Bonds
Source: Chemistry. 2025 Sep 7;31(63):e02215. doi: 10.1002/chem.202502215 (PMC12619045; doi:10.1002/chem.202502215)
Supplement: Supplementary file 1 — Supporting Information [file CHEM-31-e02215-s003.pdf]

Supporting Information

A Phosphorus(V)-Centered Porphyrin Having Redox- and Air-Stable Axial P–H Bonds

Shintaro Ishida, Yoshiki Ota, Nozomu Kuwabara, Takuroh Hatakeyama, and Takeaki Iwamoto**

Department of Chemistry, Graduate School of Science, Tohoku University

Sendai 980-8578, Japan

Email: ishida@tohoku.ac.jp, takeaki.iwamoto@tohoku.ac.jp

Contents

1. Experimental Details S2

2. NMR, EPR, IR, and UV-vis Spectra S4

3. X-Ray Analyses S11

4. Theoretical Studies S13

1. Experimental Details

General Procedures

All reactions treating air-sensitive compounds were carried out under argon or nitrogen atmosphere using a high-vacuum line, standard Schlenk techniques, or a glovebox, as well as dry and oxygen-free solvents. The ^{1}H (500 MHz), ^{13}C (126 MHz), ^{19}F (471 MHz), and ^{31}P (202 MHz) NMR spectra were recorded on a Bruker Avance III 500 FT NMR spectrometer. The ^{1}H and ^{13}C NMR chemical shifts were referenced to the ^{1}H and ^{13}C NMR signals of the residual solvents; chloroform-*d* (^{1}H δ 7.26 and ^{13}C δ 77.0). The ^{31}P NMR chemical shifts were relative to 85% H_3PO_4. (δ 0.0). The ^{19}F NMR chemical shifts were relative to C_6F_6 (in $CDCl_3$, δ –163.0). Sampling of air-sensitive compounds was carried out using a VAC NEXUS 100027 type glovebox. High-resolution mass spectra were performed on a JEOL JMS-T100GCV spectrometer using a FD+(eiFi) method. IR spectrum was recorded on a HORIBA FT-720 spectrometer. EPR spectra were recorded on a JEOL X330 EPR spectrometer. Elemental analysis was performed with a J-SCIENCE LAB JM-11.

Materials

Dry and degassed hexane and THF were prepared using a VAC 103991 solvent purifier. CH_2Cl_2 was dried over CaH_2 and then distilled prior to use by using a vacuum line. Chloroform-d_1 and pyridine were dried over molecular sieves 4Å. $POCl_3$, $LiAlH_4$, benzaldehyde, and $AgPF_6$ were commercially available and used as received. 5,10,15,20-Tetrakis(4-*tert*-butylphenyl)porphyrin **3** was prepared according to the published procedure.[47]

Synthesis of 2^{+}•Cl^{-}

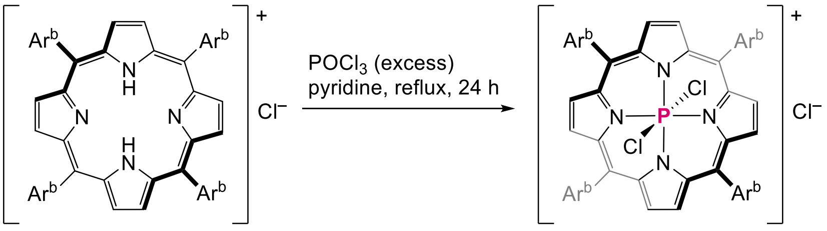

Ar^b = 4-*tert*-butylphenyl

In a two-necked flask (300 mL) equipped with a magnetic stir bar and a Dimroth condenser, free-base porphyrin **3** (1.38 g, 1.64 mmol), $POCl_3$ (ca. 15 mL), and pyridine (50 ml) were placed and refluxed for 24 h. Then, the resulting solution was cooled to room temperature and poured into a separatory funnel. CH_2Cl_2 (ca. 50 mL) was added to the separatory funnel and the reaction mixture was washed by cold water (ca. 50 mL) and then brine (ca. 50 mL). The combined organic layer was dried over anhydrous $MgSO_4$. Insoluble materials were filtered off, and the filtrate was concentrated under reduced pressure. The crude product was purified by Al_2O_3 column chromatography (eluent: CH_2Cl_2:MeOH = 50:1) and recrystallization from CH_2Cl_2/hexane provided **2**$^{+}$•Cl^{-} in 1.31 g (1.34 mmol, 82% yield) as blue crystals.

2^{+}•Cl^{-}: blue crystals; mp > 250 °C; ^{1}H NMR (500 MHz, $CDCl_3$, 296 K) δ 1.51 (s, 36H, *tert*-Bu), 7.78 (d, J = 8.0 Hz, 8H, Ar^{b}), 7.90 (d, J = 8.0 Hz, 8H, Ar^{b}), 9.13 (brs, 8H, β-pyrrole); ^{13}C NMR (126 MHz, $CDCl_3$, 298 K) δ 31.4 (C*Me*$_3$), 35.1 (*C*Me$_3$), 117.8 (C, d, $^{3}J_{PC}$ = 2.9 Hz, *meso*), 125.7 (CH, *m*-Ar^{b}), 131.4 (C, *ipso*-Ar^{b}), 132.5 (CH, d, $^{3}J_{PC}$ = 6.6 Hz, β-pyrrole), 133.1 (CH, *o*-Ar^{b}), 139.9 (C, α-pyrrole), 153.6 (C, *p*-Ar^{b}); ^{31}P{^{1}H} NMR (202 MHz, $CDCl_3$, 297 K) δ –227.9; HRMS (FD) *m*/*z*: **2**$^{+}$ calcd for $C_{60}H_{60}Cl_2N_4P$ 937.3933; Found 937.3928; UV-vis (THF) λ_{max}/nm (ε) 448 (5.9×10^{4}), 575 (3.0×10^{3}), 622 (2.9×10^{3}); Anal. Calcd for $[C_{60}H_{60}Cl_2N_4P]^{+}Cl^{-}\cdot H_2O$: C, 72.61; H, 6.30; N, 5.65%. Found: C, 72.75; H, 6.34; N, 5.53%.

Synthesis of $1^{+}PF_6^{-}$

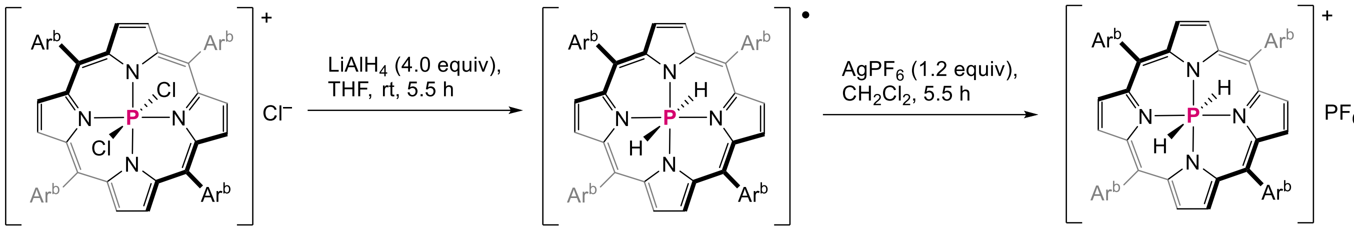

$\mathbf{2}^{+}$•Cl⁻ $\mathbf{1}^{\bullet}$ $\mathbf{1}^{+}$•PF_6^{-}

Ar^b = 4-*tert*-butylphenyl

In a Schlenk-tube (200 mL) equipped with a magnetic stir bar, **2**⁺•Cl⁻ (300 mg, 0.308 mmol), $LiAlH_4$ (47 mg, 1.23 mmol), and THF (44 mL) were placed and stirred at room temperature in the dark for 5.5 h. The color of solution turned to reddish yellow during the reaction. The insoluble materials were filtered off through a pad of celite to remove the residual aluminum hydride species, and the volatiles were removed in vacuo. Then, CH_2Cl_2 (ca. 60 mL) was added and then the insoluble materials were filtered off through a pad of celite. After the volatiles were removed in vacuo, a crude product (277 mg) containing $\mathbf{1}^{\bullet}$ was obtained. In a Schlenk-tube (200 mL) equipped with a magnetic stir bar, the crude product, $AgPF_6$ (977 mg, 0.383 mmol), and CH_2Cl_2 (58 mL) were placed and stirred for 5.5 h in the dark. The color of the solution turned green during the reaction. The insoluble materials were filtered off through a pad of celite, and the volatiles were removed in vacuo. The product was purified by silica-gel column chromatography (eluent: toluene, then CH_2Cl_2) and recrystallization from CH_2Cl_2 with hexane (solvent diffusion method, v/v = ca. 1:3) afforded **1**⁺•PF_6^{-} (125 mg, 0.123 mmol) in 40% as blue crystals. The yields of **1**⁺•PF_6^{-} vary from 20% to 40% in the same reaction conditions.

1⁺•PF_6^{-}: blue crystals; mp > 250 °C; ^{1}H NMR (500 MHz, $CDCl_3$, 295 K) δ −2.19 (d, 2H, $^{1}J_{PH}$ = 1023 Hz, PH) 1.53 (s, 36H, *tert*-Bu), 7.76 (d, J = 8.0 Hz, 8H, Ar^b), 7.88 (d, J = 8.0 Hz, 8H, Ar^b), 9.07 (s, 8H, β-pyrrole); ^{13}C NMR (126 MHz, $CDCl_3$, 296 K); δ 31.5 (C*Me*$_3$), 35.1 (*C*Me$_3$), 116.3 (C, *meso*), 125.4 (CH, *m*-Ar^b), 133.0 (C, *ipso*-Ar^b), 133.4 (CH, β-pyrrole), 133.9 (CH, *o*-Ar^b), 139.6 (C, α-pyrrole), 152.9 (C, *p*-Ar^b);^{19}F NMR (471 MHz, $CDCl_3$, 295 K); δ 74.5 (d, $^{1}J_{PF}$ = 712 Hz, PF_6); ^{31}P NMR (202 MHz, $CDCl_3$, 295 K) δ −251.4 (t, $^{1}J_{PH}$ = 1023 Hz, PH_2), −144.1 (sept, $^{1}J_{PF}$ = 713 Hz, PF_6); ^{31}P{^{1}H} NMR (202 MHz, $CDCl_3$, 295 K) δ −251.2 (s, PH_2), −144.2 (sept, $^{1}J_{PF}$ = 713 Hz, PF_6); HRMS(FD) *m*/*z*: **1**⁺ calcd for $C_{60}H_{62}N_4P$ 869.4714; Found 869.4714; UV-vis (THF) λ_{max}/nm (ε) 451 (7.2×10^4), 587 (2.7×10^3), 633 (3.7×10^3); Anal. Calcd for $[C_{60}H_{62}N_4P]^+[PF_6]^-$: C, 70.99; H, 6.16; N, 5.52%. Found: C, 71.01; H, 6.32; N, 5.60%.

Cyclic Voltammetry

The CV cell consisted of a glassy carbon working electrode, a Pt wire counter electrode, and an $Ag/AgNO_3$ reference electrode. The CV measurements of **2**⁺•Cl⁻ and **1**⁺•PF_6^{-} were carried out under argon atmosphere in a dry THF solution at room temperature. Tetrabutylammonium hexafluorophosphate ($Bu_4N^+PF_6^-$) was used as a supporting electrolyte (0.1 M). The redox potentials are referenced to the ferrocene/ferrocenium ion couple.

2. NMR, EPR, IR, and UV-vis spectra

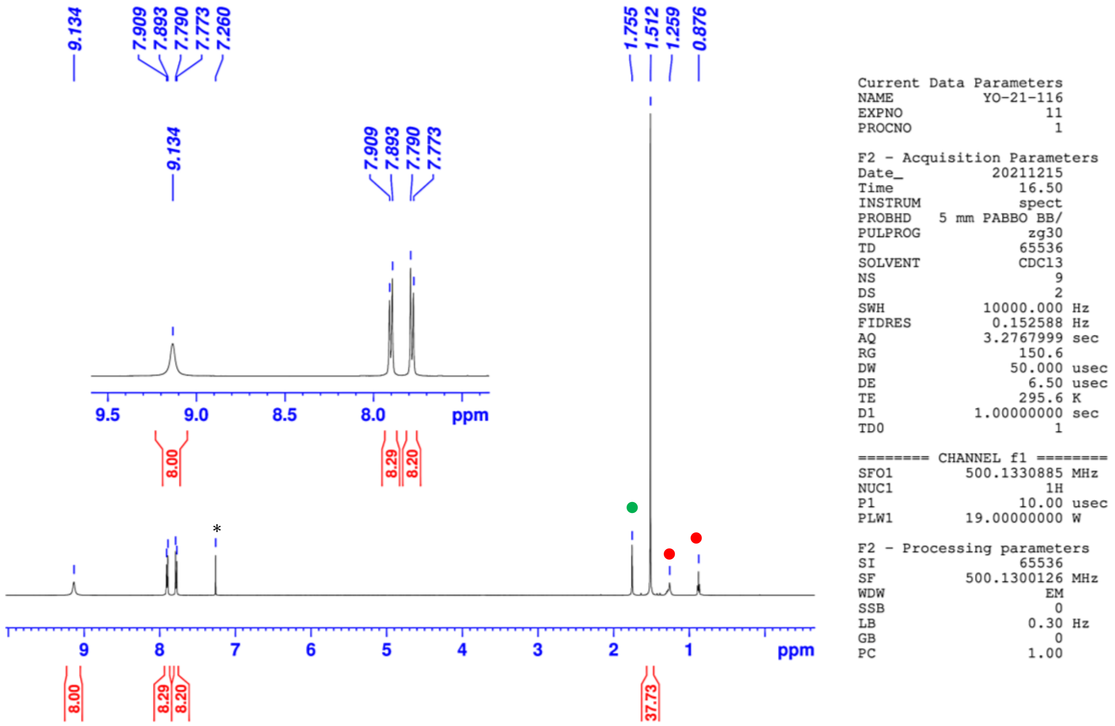

Figure S1. ^{1}H NMR spectrum of **2**$^{+}$•Cl^{-} in $CDCl_3$ at 296 K (* = $CHCl_3$, ● = hexane, ● = H_2O).

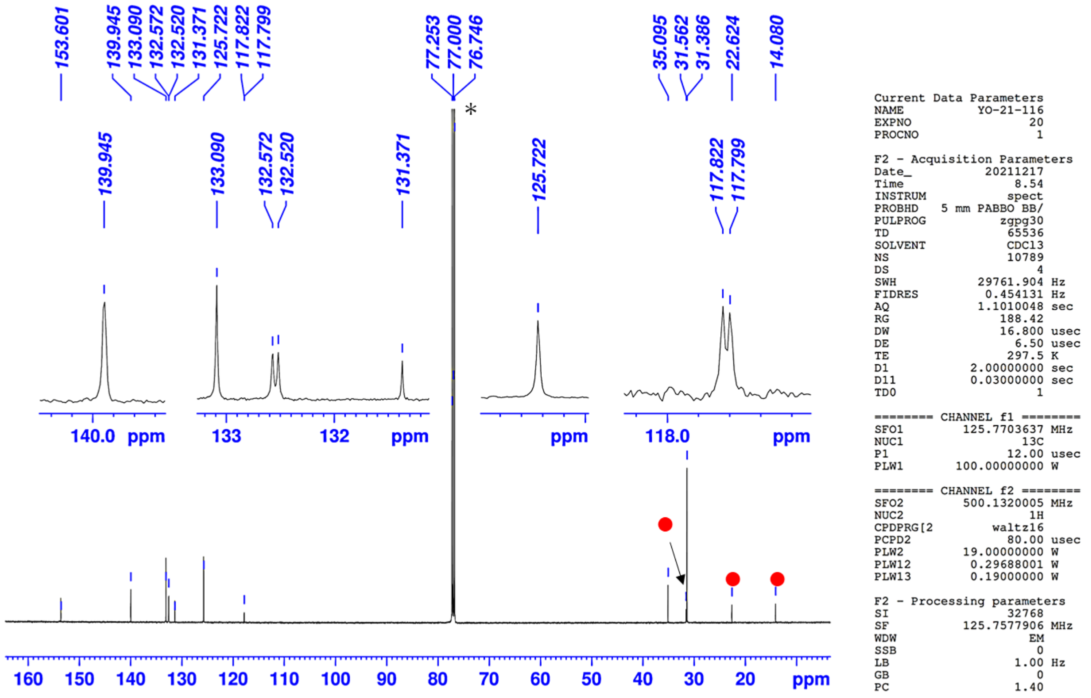

Figure S2. ^{13}C{^{1}H} NMR spectrum of **2**$^{+}$•Cl^{-} in $CDCl_3$ at 298 K (* = $CDCl_3$, ● = hexane).

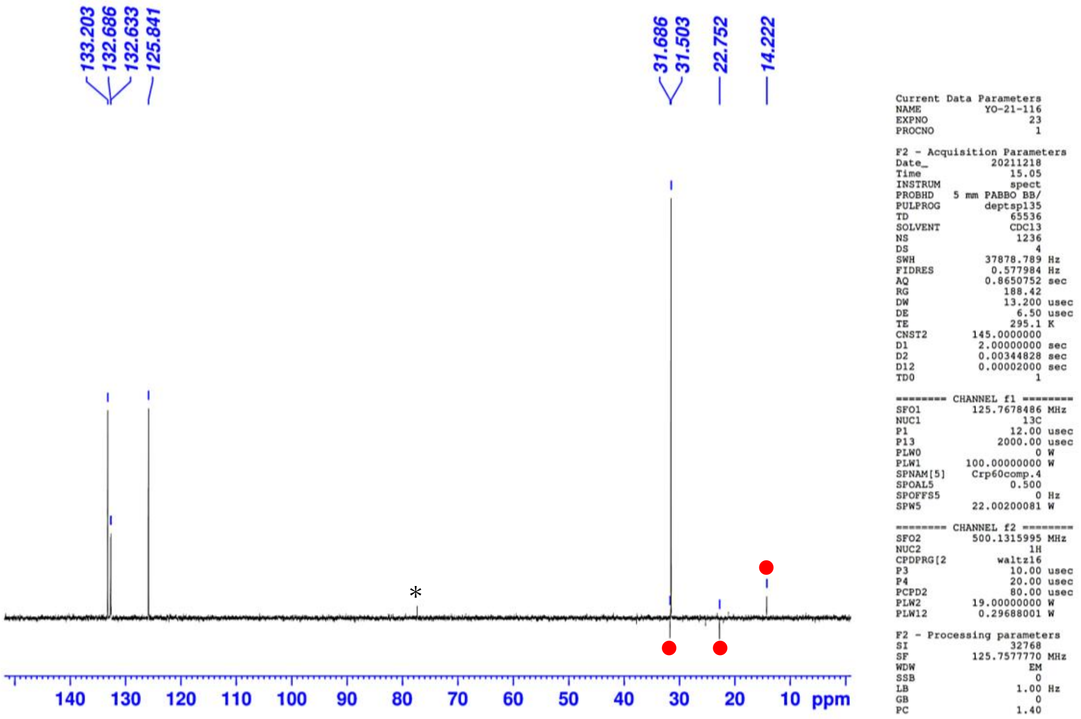

Figure S3. $^{13}C\{^1H\}$ (DEPT 135) NMR spectrum of **2**$^+$•Cl^- in $CDCl_3$ at 295 K (* = $CHCl_3$, ● = hexane).

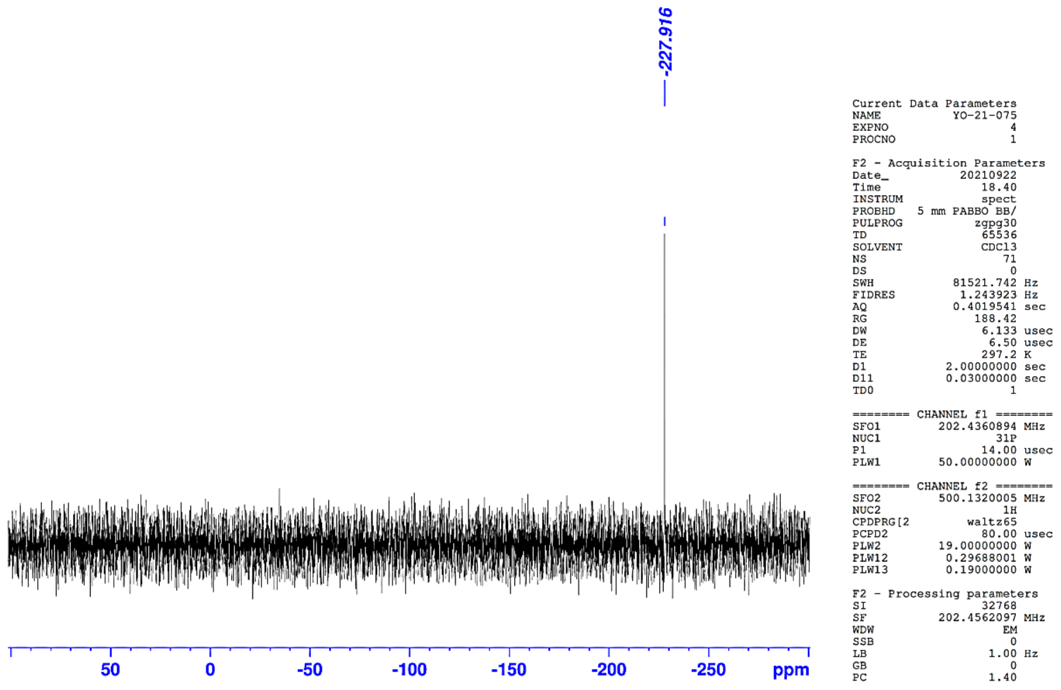

Figure S4. $^{31}P\{^1H\}$ NMR spectrum of **2**$^+$•Cl^- in $CDCl_3$ at 297 K.

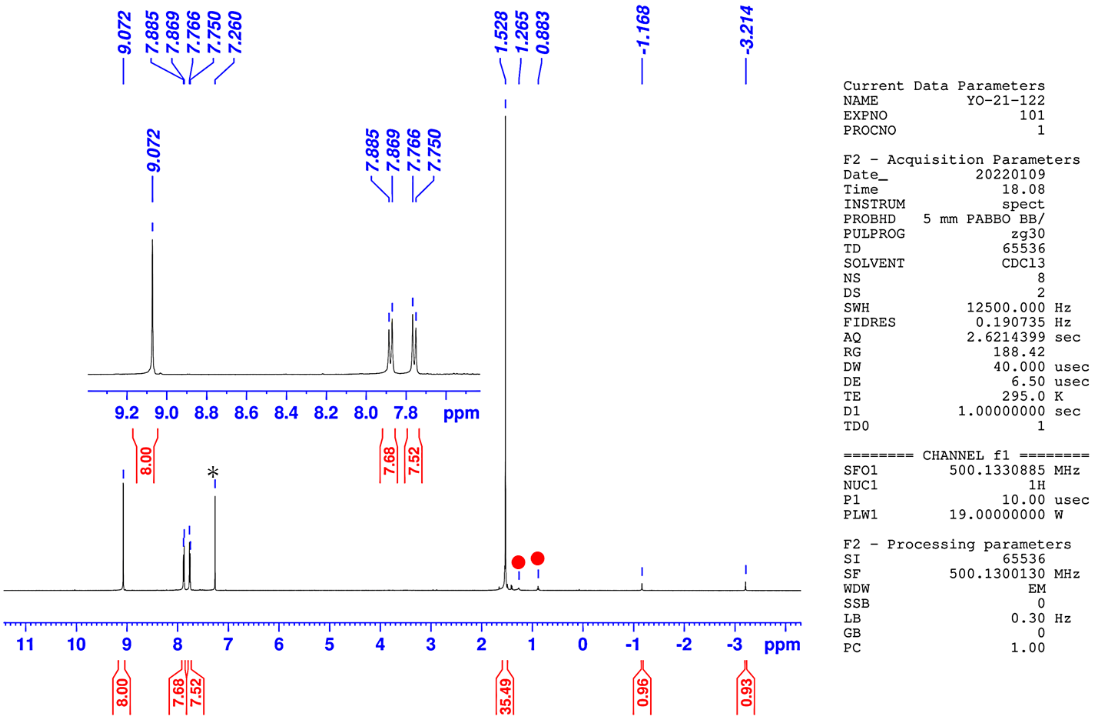

Figure S5. ^{1}H NMR spectrum of **1**$^{+}$•PF_6^{-} in $CDCl_3$ at 295 K (* = $CHCl_3$, ● = hexane).

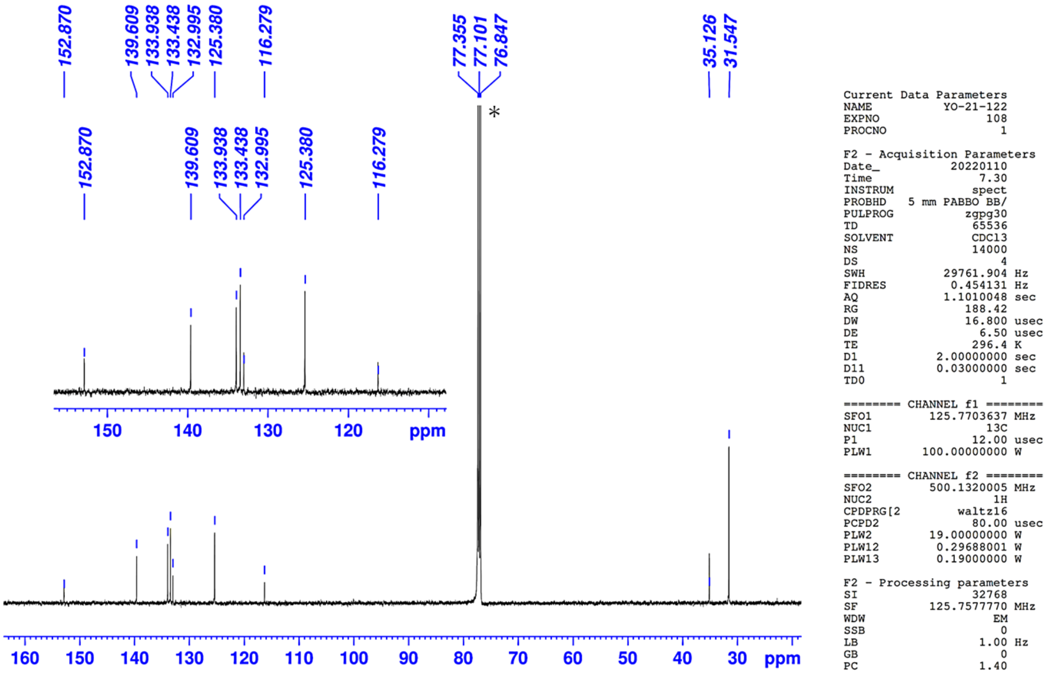

Figure S6. $^{13}C\{^{1}H\}$ NMR spectrum of **1**$^{+}$•PF_6^{-} in $CDCl_3$ at 296 K (* = $CDCl_3$).

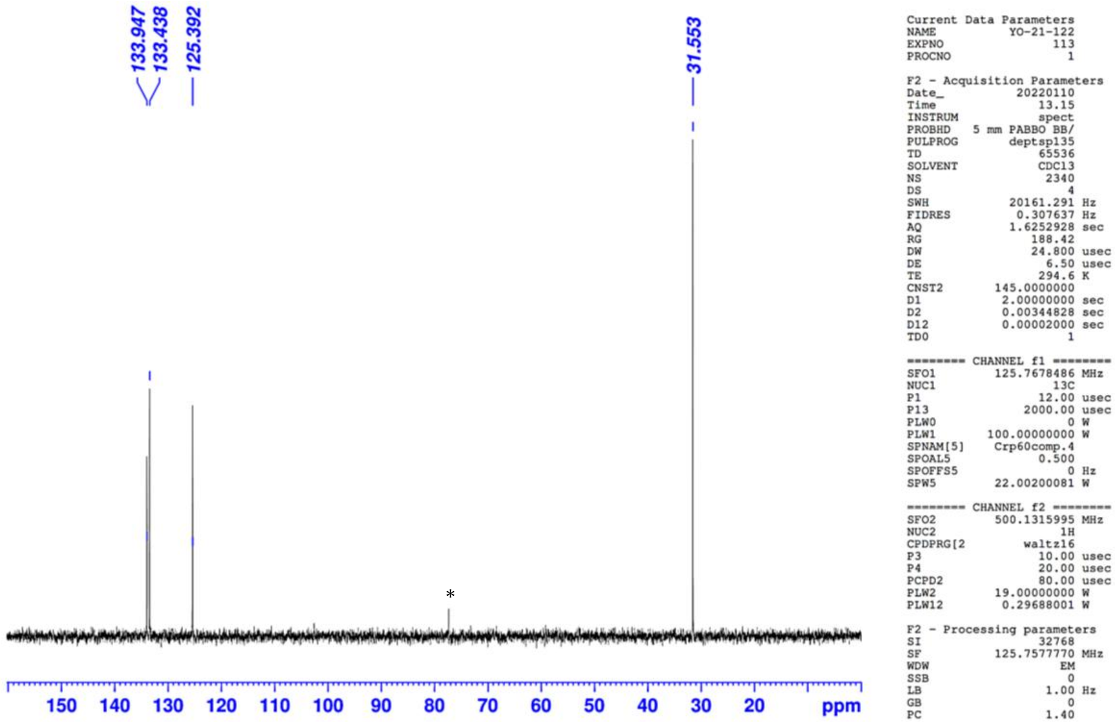

Figure S7. $^{13}C\{^{1}H\}$ (DEPT 135) NMR spectrum of **1**$^{+}$•PF_6^{-} in $CDCl_3$ at 295 K (* = $CHCl_3$).

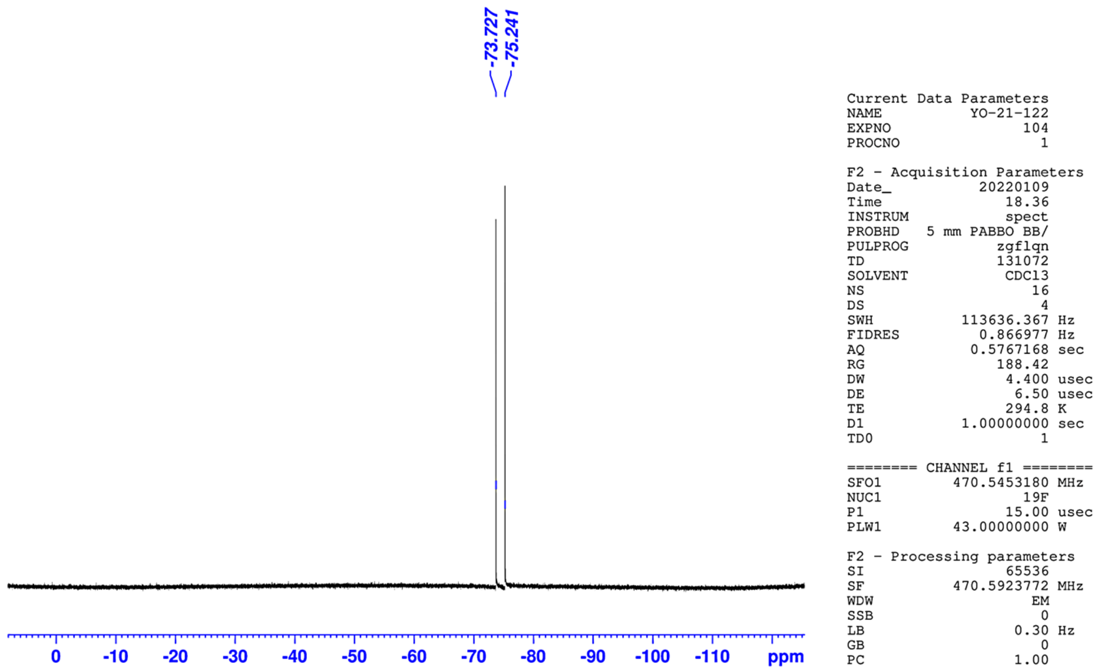

Figure S8. ^{19}F NMR spectrum of **1**$^{+}$•PF_6^{-} in $CDCl_3$ at 295 K.

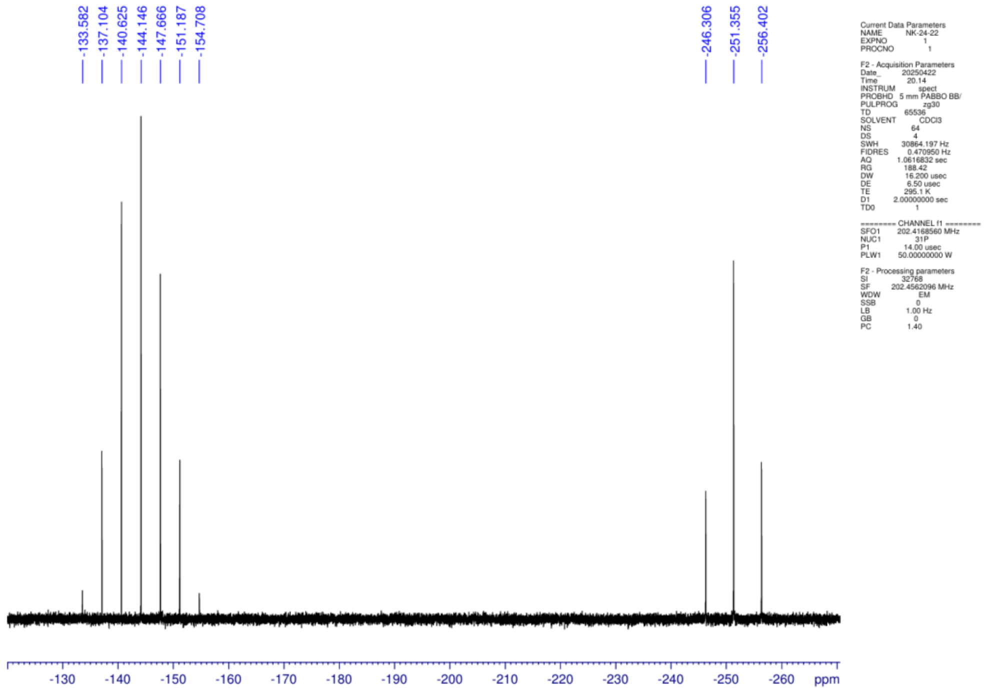

Figure S9. ^{31}P NMR spectrum of **1**$^{+}$•PF_6^{-} in $CDCl_3$ at 295 K.

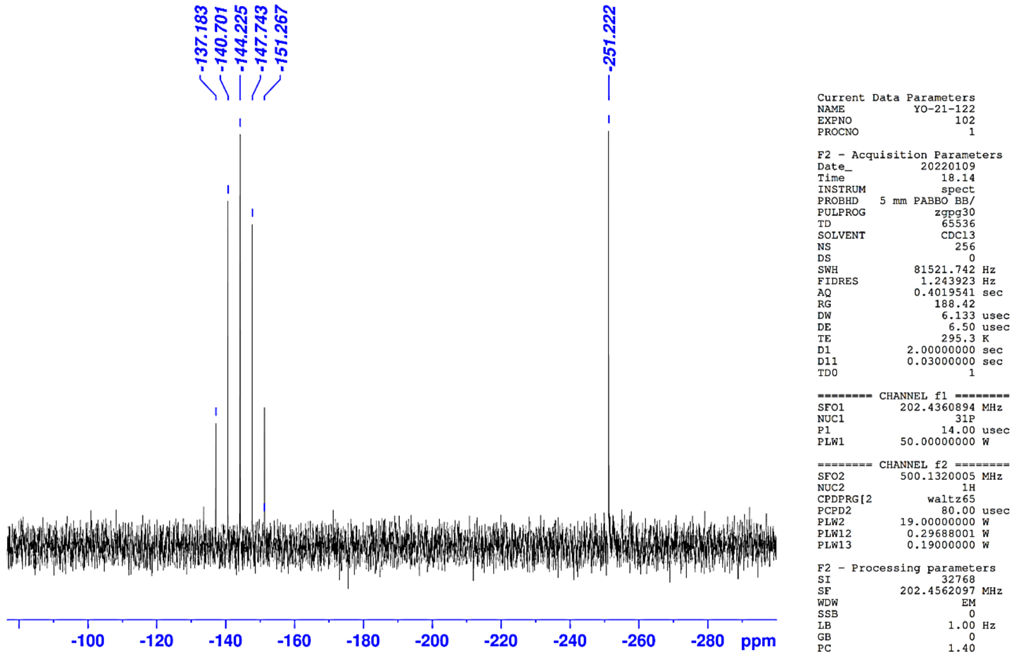

Figure S10. ^{31}P{^{1}H} NMR spectrum of **1**$^{+}$•PF_6^{-} in $CDCl_3$ at 295 K. The outermost peaks of the septet signal were missing due to the low digital resolution.

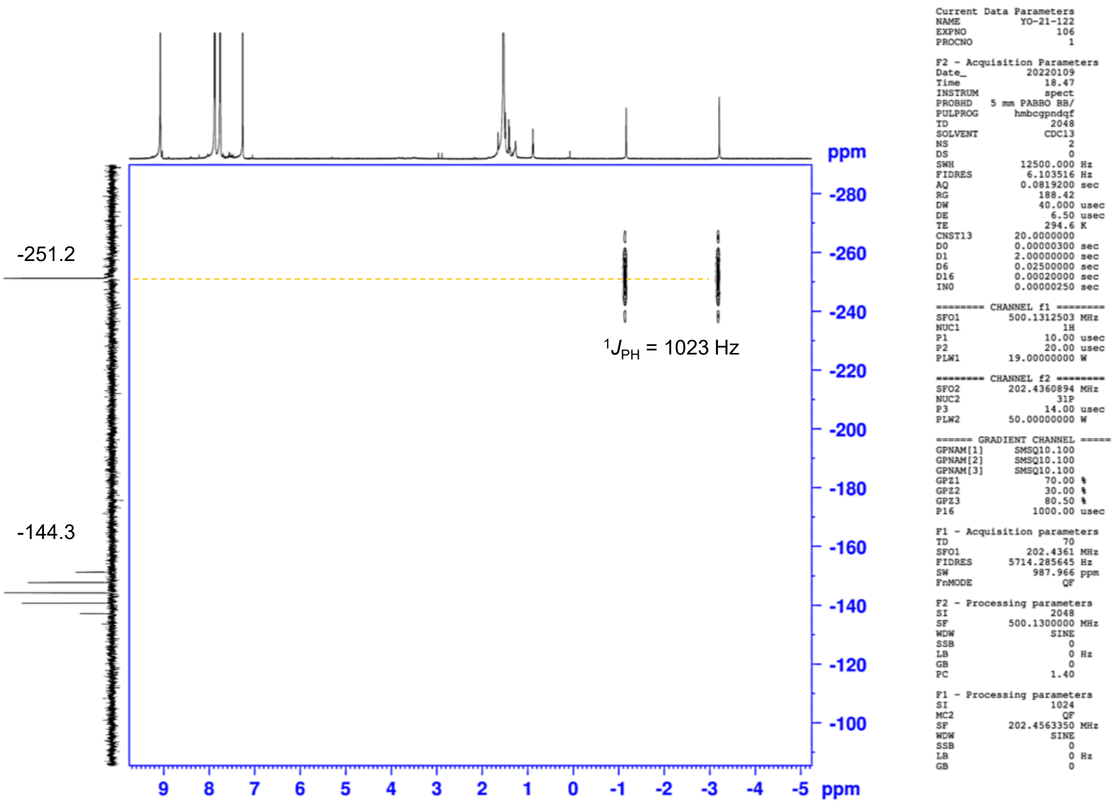

Figure S11. ^{1}H-^{31}P HMBC NMR spectrum of **1**$^+$•PF_6^- in $CDCl_3$ at 295 K.

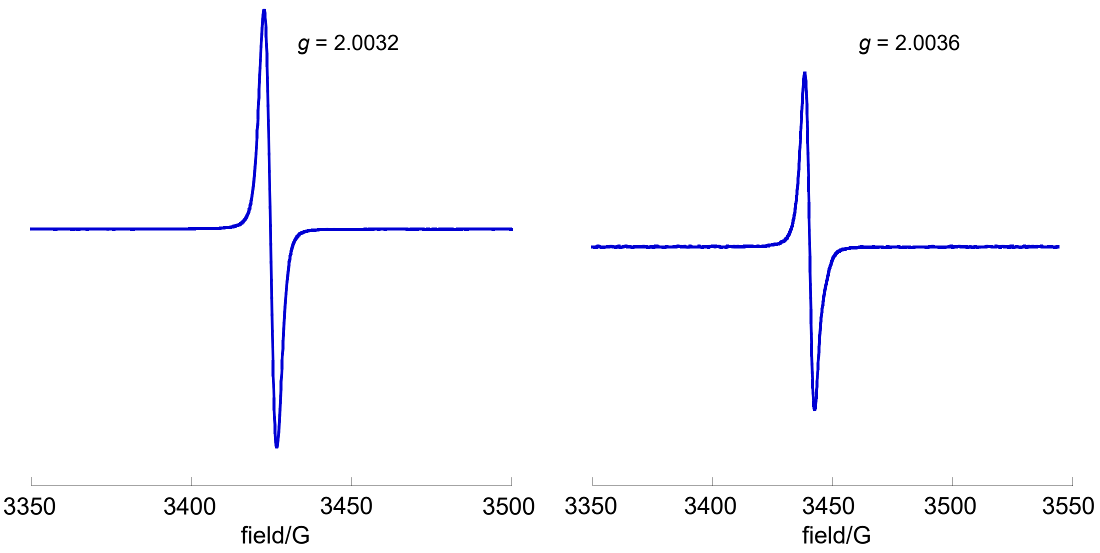

Figure S12. EPR spectra of the reaction mixture of **2**$^+$•Cl^- and $LiAlH_4$ in THF at room temperature (left) and the reaction mixture of **1**$^+$•PF_6^- with KC_8 (1.0 equiv.) in C_6D_6 solution at room temperature (right).

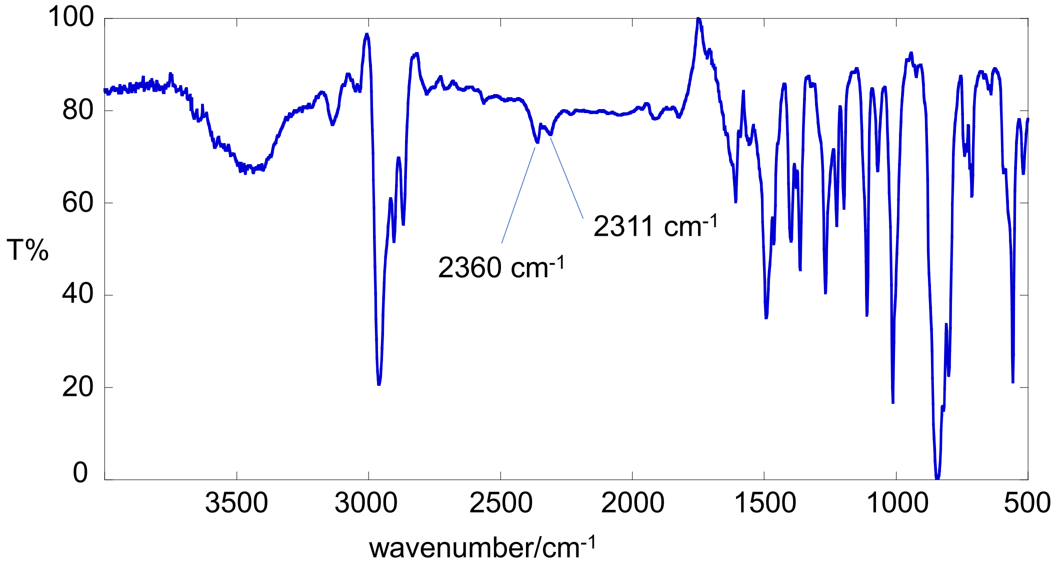

Figure S13. IR spectrum of **1**$^+$•PF_6^- (KBr pellet, rt). The absorption band at 2360 cm^{-1} was gradually decreased during the measurements, while that at 2311 cm^{-1} is almost unchanged. The symmetric stretching mode of H–P–H moiety is IR-forbidden, while the corresponding asymmetric mode is IR-allowed. In addition, the DFT study of the model compounds (**1m**$^+$) indicated that the intensity of the symmetric stretching mode of H–P–H moiety was zero. Hence, the band at 2360 cm^{-1} is assignable to the asymmetric stretching mode of H–P–H moiety [ν_{as}(PH)]. The band at 2311 cm^{-1} would be a decomposed product such as a partially hydrolyzed P-porphyrin.

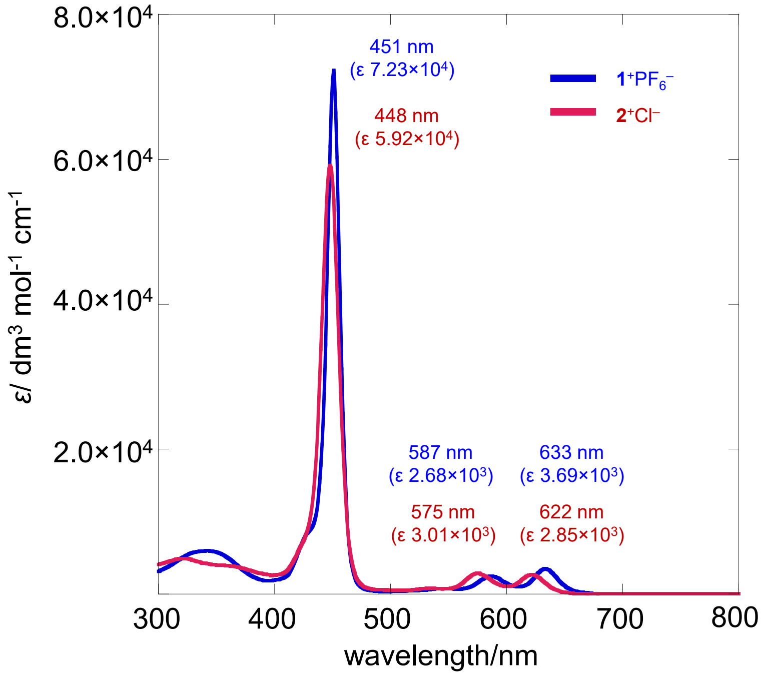

Figure S14. UV-vis spectra of **1**$^+$•PF_6^- and **2**$^+$•Cl^- in THF at room temperature.

3. X-Ray Analyses

Single crystals suitable for X-ray diffraction study were obtained (1) from a saturated CH_2Cl_2 solution (layering: hexane/CH_2Cl_2) at –25 °C for **2**$^+$•Cl^- and (2) from a saturated chlorobenzene solution with hexane at room temperature by a slow vapor diffusion technique for **1**$^+$•PF_6^-. The single crystals coated by Apiezon grease were mounted on a glass fiber and transferred to the cold nitrogen gas stream on the diffractometer. X-ray data were collected on a Rigaku XtaLAB AFC10 diffractometer with a HyPix-6000HE hybrid pixel array detector with graphite monochromated Mo-Kα radiation (λ 0.71073 Å). The data were corrected for Lorentz and polarization effects. An empirical absorption correction based on the CrysAlisPro 1.171.40.61a (Rigaku Oxford Diffraction, 2019) using spherical harmonics, implemented in SCALE3 ABSPACK scaling algorithm. The structure was solved by direct method and refined by full-matrix least squares against F^2 using all data (SHELXL-2018).[67][68] Molecular structure was analyzed by Yadokari-XG software.[69] The single crystal **2**$^+$$Cl^-$ contained highly disordered chlorides and solvent molecules (water and CH_2Cl_2). As the positions of water were hardly determined by the Fourier map and the water molecules were treated by the SQUEEZE procedure in PLATON.[70]

Crystallographic data of **2**$^+$•Cl^- (90 K) [CCDC: 2262176]: $C_{62}H_{64}Cl_7N_4P$; Mw 1144.29; *Triclinic*; space group *P*-1, a = 12.1957(2) Å, b = 15.1443(2), c = 18.2251(3) Å, α = 96.3340(10)°, β = 108.8550(10)°, γ = 103.4470(10)°, V = 3034.57(8) Å^3, Z = 2, D_{calcd} = 1.252 Mg/m^3, $R1$ = 0.0614, ($I > 2\sigma(I)$), $wR2$ = 0.1669 (all data), GOF = 1.040.
Crystallographic data of **1**$^+$•PF_6^- (120 K) [CCDC: 2262175]: $C_{66}H_{67}ClF_6N_4P_2$; Mw 1127.62; *Monoclinic*; space group *C*2/*c*, a = 25.6310(4) Å, b = 15.88557(3), c = 44.5088(8) Å, β = 92.5148(16)°, V = 18105.0(6) Å^3, Z = 12, D_{calcd} = 1.241 Mg/m^3, $R1$ = 0.0867, ($I > 2\sigma(I)$), $wR2$ = 0.2304 (all data), GOF = 1.074.

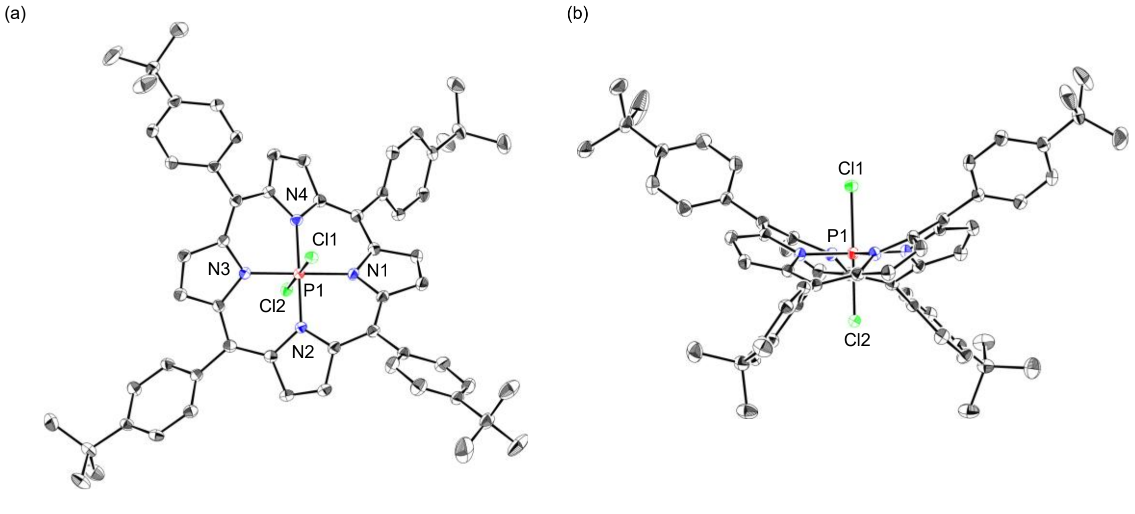

Figure S15. ORTEPs of **2**$^+$•Cl^-. Hydrogen atoms, counter anions, and solvent molecules (CH_2Cl_2) were omitted for clarity. Thermal ellipsoids were drawn at 50% probability level. (a) Top view and (b) side view.

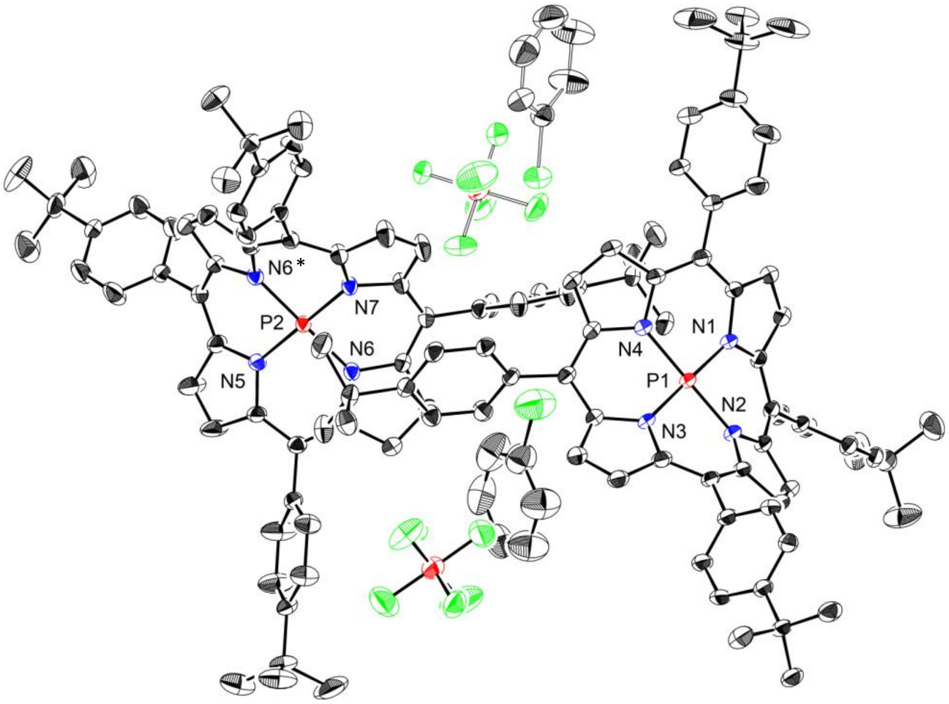

Figure S16. An ORTEP of an asymmetric unit of **1**$^+$•$PF_6^–$. The asymmetric unit contains two crystallographically independent **1**$^+$ molecules (mol-A and mol-B) and two chlorobenzenes and $PF_6^–$. The N5–P2–N7 moiety in the mol- B is located on the crystallographically two-fold axis. Hydrogen atoms were omitted for clarity.

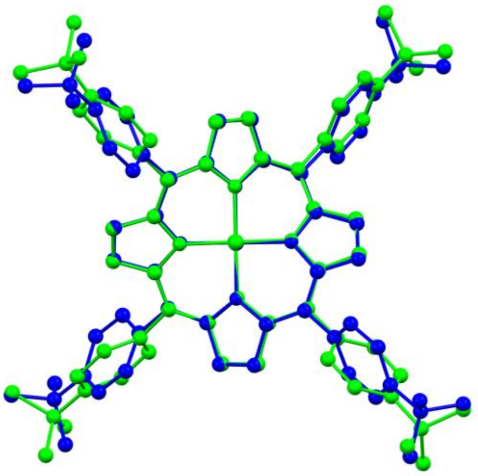

Figure S17. Superimposed structures of mol-A (green) and mol-B (blue) of **1**$^+$•$PF_6^–$. Hydrogen atoms are omitted for clarity. The metric parameters of the porphyrin core between mol-A and mol-B are essentially identical, while some orientations of aryl rings are different.

4. Theoretical Studies

All theoretical calculations were performed using Gaussian 09,[61] GRRM 14,[62][71][72] NBO7.0,[63] and Multiwfn[73][74] programs. Geometry optimization and frequency analysis of **1m**$^+$ were carried out at the B3PW91-D3/6-31+G(d) level of theory. No imaginary frequency was found in the equilibrium structure. The calculated Cartesian coordinates and energies of **1m**$^+$ are tabulated in Table S1. Chemical shifts of **1m**$^+$ were calculated using a GIAO method at the B3PW91/6-311+G(2df,p) level of theory. PH_3 was used for a standard of ^{31}P NMR chemical shifts.

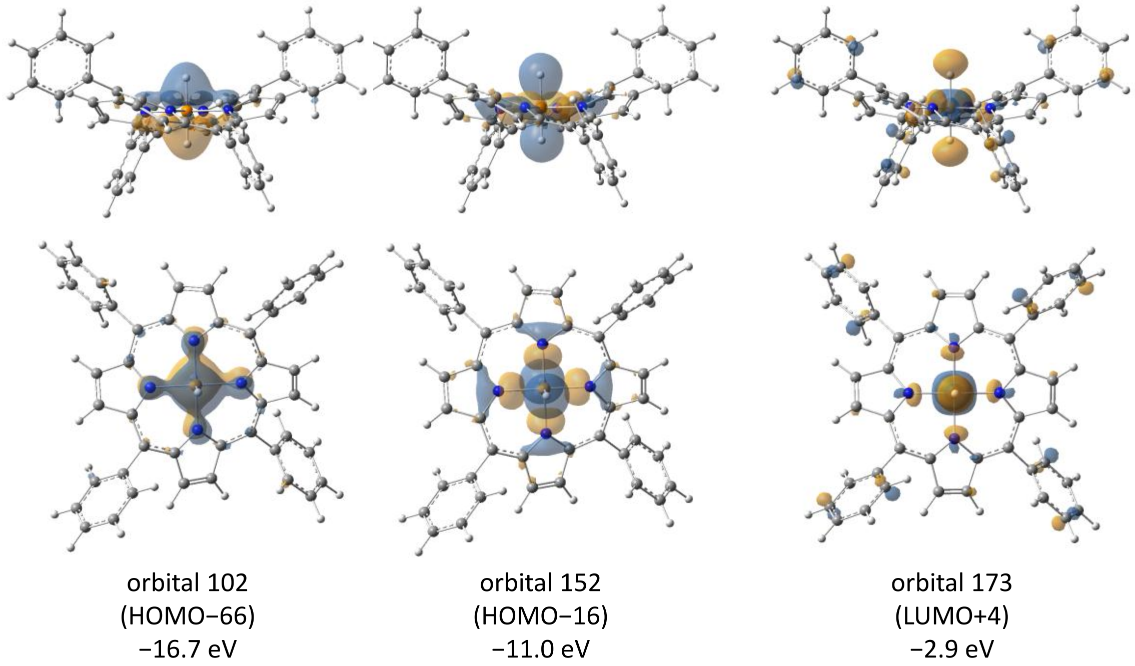

Figure S18. Selected Kohn-Sham orbitals of **1m**$^+$ calculated at the B3PW91-D3/6-31+G(d) level of theory.

Table S1. Atomic Coordinates of **1m**$^+$

Atomic Type	X	Y	Z
P	0.000000711959	3.848728643106	8.486685652581
N	-0.918889458650	2.203163863850	8.482328912583
C	-0.472419090120	1.012266863829	9.024312621957
C	0.861666098681	0.754705786642	9.354269942990
C	1.862272730403	1.651324197118	8.958263801708
C	3.223244859738	1.289103299228	8.708045100908
H	3.657461002932	0.332259343396	8.964465295319
C	3.802820230476	2.324347136006	8.028406951696
H	4.809885737029	2.381086822336	7.639069528837
C	1.271768768591	-0.548166039625	9.934621560393
C	1.097928730929	-1.748203467333	9.230667511028
H	0.657237273417	-1.728329917496	8.236868633140
C	1.518998836096	-2.955746066721	9.784503594674
H	1.387410599520	-3.879097314778	9.226544439799
C	2.117524493502	-2.977779394224	11.045394115826
H	2.443769171757	-3.920345138208	11.476704706146
C	2.303328498054	-1.786072210228	11.747268203045
H	2.771468275480	-1.797227278252	12.728005506598
C	1.889033023222	-0.576082605897	11.192836407543
H	2.033116331136	0.353608219822	11.738266081502
H	0.000032381150	3.848714097601	7.080589927948
N	0.918891280235	5.494293098590	8.482336256340
C	0.472396774409	6.685201418405	9.024274993973
C	-0.861702844436	6.942769130109	9.354168366005
C	-1.862292065793	6.046142712624	8.958136920077
C	-3.223253570586	6.408357887523	8.707852878323
H	-3.657480863758	7.365206728500	8.964235821233
C	-3.802799406297	5.373100075316	8.028210889550
H	-4.809848523157	5.316351711371	7.638832349670
C	-1.271830476577	8.245653037807	9.934475744109
C	-1.097968019631	9.445675088540	9.230501113387
H	-0.657238978288	9.425780383932	8.236719238302
C	-1.519062294743	10.653228879979	9.784294699878
H	-1.387456331844	11.576568085805	9.226319679884
C	-2.117633997719	10.675288659551	11.045162996281
H	-2.443897465892	11.617863151843	11.476440393668
C	-2.303459377624	9.483596711240	11.747057340473
H	-2.771634777404	9.494772389766	12.727777436487
C	-1.889139902779	8.273595885299	11.192667811863
H	-2.033239862621	7.343916864743	11.738113301157
N	-1.645563987204	4.767619121851	8.490992743205
C	-2.836454775389	4.321130998133	7.949010283894
C	-3.094011923078	2.987035248433	7.619092841158
C	-2.197398150964	1.986441394951	8.015141317213
C	-2.559621826414	0.625477215672	8.265398820633
H	-3.516462619447	0.191252926208	8.008980676685
C	-1.524386107854	0.045923585224	8.945067848602
H	-1.467651218801	-0.961129538946	9.334438010530
C	-4.396877692297	2.576914622630	7.038739969385
C	-5.596921726892	2.750772063796	7.742678485589
H	-5.577057628483	3.191490908221	8.736465464394
C	-6.804458847474	2.329685499377	7.188842741309
H	-7.727815440641	2.461287507220	7.746789949659
C	-6.826479932872	1.731126408139	5.927967808306
H	-7.769041406916	1.404868980965	5.496657359026

C	-5.634766099401	1.545305610063	5.226109279210
H	-5.645911668645	1.077139905295	4.245384204332
C	-4.424781945969	1.959617389894	5.780541046126
H	-3.495085913343	1.815521093947	5.235123550619
H	-0.000030787039	3.848743225131	9.892781371960
N	1.645565323329	2.929838446811	8.491084685083
C	2.836479548640	3.376315081772	7.949144034240
C	3.094051028544	4.710404092580	7.619210320418
C	2.197420209935	5.711006124279	8.015199919588
C	2.559632529725	7.071975740311	8.265444491963
H	3.516484408941	7.506194885646	8.009059036275
C	1.524366642108	7.651543554752	8.945055507163
H	1.467613388710	8.658605273876	9.334400780069
C	4.396940139265	5.120513737636	7.038902403721
C	5.596956760270	4.946668114367	7.742890389828
H	5.577054179163	4.505966270488	8.736684078732
C	6.804515223992	5.367745821156	7.189094543885
H	7.727850034022	5.236153265667	7.747079853581
C	6.826585262236	5.966284423734	5.928210815710
H	7.769163368479	6.292535065644	5.496931760686
C	5.634898783705	6.152093557222	5.226303032473
H	5.646082382983	6.620243282699	4.245570822804
C	4.424893351873	5.737790517949	5.780694515292
H	3.495218632425	5.881877836055	5.235238509965

Symmetry: S4

Free Energy (298.150 K and 1.000 Atm) = -2253.729657439545 au
